# Slightly increased BMI at young age is a risk factor for future hypertension in Japanese men

**DOI:** 10.1371/journal.pone.0191170

**Published:** 2018-01-11

**Authors:** Yuki Someya, Yoshifumi Tamura, Yoshimitsu Kohmura, Kazuhiro Aoki, Sachio Kawai, Hiroyuki Daida

**Affiliations:** 1 Sportology Center, Juntendo University Graduate School of Medicine, Tokyo, Japan; 2 Department of Metabolism & Endocrinology, Juntendo University Graduate School of Medicine, Tokyo, Japan; 3 Faculty of Health and Sports Science, Juntendo University, Chiba, Japan; 4 Juntendo University Graduate School of Health and Sports Science, Chiba, Japan; 5 Department of Cardiology, Juntendo University Graduate School of Medicine, Tokyo, Japan; Universita degli Studi di Ferrara, ITALY

## Abstract

**Background:**

Hypertension is developed easily in Asian adults with normal body mass index (BMI) (~23 kg/m^2^), compared with other ethnicities with similar BMI. This study tested the hypothesis that slightly increased BMI at young age is a risk factor for future hypertension in Japanese men by historical cohort study.

**Methods:**

The study participants were 636 male alumni of the physical education school. They had available data on their physical examination at college age and follow-up investigation between 2007 and 2011. The participants were categorized into six categories: BMI at college age of <20.0 kg/m^2^, 20.0–21.0kg/m^2^, 21.0–22.0kg/m^2^, 22.0–23.0kg/m^2^, 23.0–24.0kg/m^2^, and ≥24.0kg/m^2^, and the incidence of hypertension was compared.

**Results:**

This study covered 27-year follow-up period (interquartile range: IQR: 23–31) which included 17,059 person-years of observation. Subjects were 22 (22–22) years old at graduated college, and 49 (45–53) years old at first follow-up investigation. During the period, 120 men developed hypertension. The prevalence rates of hypertension for lowest to highest BMI categories were 9.4%, 14.6%, 16.1%, 17.5%, 30.3%, and 29.3%, respectively (p<0.001 for trend), and their hazard ratios were 1.00 (reference), 1.80 (95%CI: 0.65–4.94), 2.17 (0.83–5.64), 2.29 (0.89–5.92), 3.60 (1.37–9.47) and 4.72 (1.78–12.48), respectively (p<0.001 for trend). This trend was similar after adjustment for age, year of graduation, smoking, current exercise status and current dietary intake.

**Conclusion:**

Slightly increased BMI at young age is a risk factor for future hypertension in Japanese men.

## Introduction

Overweight, defined by a body mass index (BMI) of 25.0 kg/m^2^ or more, is recognized as a risk factor for mortality[1, 2], cardiovascular disease (CVD) [3, 4] and metabolic disorders, such as hypertension, type 2 diabetes, and dyslipidemia [5, 6]. In addition, it has been reported that overweight and obesity (BMI >30 kg/m^2^) at ~20 years of age are also risk factors for development of hypertension in middle aged Caucasians [7, 8]. For example, overweight and obesity at ~18 years of age increase the risk of hypertension at 25–45 years of age in Caucasians [9].

In contrast, in Asian countries, the prevalence of overweight subjects at young age is very low and most of patients with hypertension have normal weight [10]. However, metabolic disorders such as hypertension, type 2 diabetes, and dyslipidemia, and cardiovascular disease are easily developed in middle aged Asian with normal BMI, compared with BMI matched other ethnicities [11–13]. Thus, slightly increased BMI level (>23 kg/m^2^) in middle age (around 50 years old) is recognized as a risk for development of hypertension in Asians [13–15]. However, it is still unclear whether slightly elevated BMI at young age within normal BMI range (<25 kg/m^2^) also increases the risk of hypertension later in life.

Only two studies addressed this research question. Heianza et al. reported that a slight increase in BMI (~1 kg/m^2^) within the normal range at 20 years of age increased the risk of future hypertension in Japanese[16]. In their health study on nurses, Huang et al. also showed that a slight increase in BMI at 18 years of age even within the normal range was positively associated with future hypertension[17]. Thus, only a few longitudinal studies investigated whether the normal BMI at young age is a risk factor for future hypertension and more studies are required to confirm this association.

In the present study, we hypothesized that the BMI at young age is associated with the development of hypertension later in life. The study was historical cohort study of the Juntendo University Alumni Study, subjects were also participated in a similar study published previously by our group [18–20]. We used standardized physical examination data of the alumni recorded when they were college students, and medical examination of self-administered questionnaires at follow-up investigation to test the study hypothesis.

## Methods

### Study participants

The Juntendo University Alumni Study included male alumni of the Physical Education School of Juntendo University. In this cohort, we demonstrated previously that association between physical fitness and physical characteristics, such as cardiorespiratory fitness, at a young age and future disease [18–20]. The majority of the study subjects engaged in college sports clubs, such as track and field, gymnastics, soccer, and judo, and participated in training for competitions in their respective sport, and were competitive athletes at least while attending university. Between 2007 and 2011, 1,385 male alumni who participated at least once follow-up investigation were studied. This study excluded the female alumni because of small percentage before 1991. Of these, 37 male alumni did not answer appropriately the questionnaire and were therefore excluded. Five hundred and fifty-three alumni who graduated before the standardized physical examination were excluded from this analysis. Thus, we finally included 636 male alumni in the current analysis.

The study protocol was approved by the Juntendo University Ethics Committee in 2007 (No.19-131). All data were anonymized before analysis to ensure privacy of the participants. Along with the questionnaire, we sent a letter of informed consent approving the collection and use of the athletic test data for research purpose. This study was carried out in accordance with the principles outlined in the Declaration of Helsinki.

### Assessment of BMI at college age

This college undergo standardized medical and physical examinations annually since 1971. We used the height and body weight recorded in the physical examination conducted at the college student. Then, average BMI during college student was calculated as weight in kilograms divided by the square of height in meters (kg/m^2^) and used for each analysis.

### Investigation for prevalence of hypertension

Incidence cases of hypertension were obtained from self-administered questionnaire between 2007 and 2011. In the survey, alumni were asked about their medical history whether they had ever been diagnosed with any disease, including hypertension, by a physician after graduation from college. Alumni were also asked about their current life-style, such as exercise, diet and smoking habits [18–20].

### Statistical analysis

For primary analysis, the participants were categorized for six categories (BMI at college age of <20.0 kg/m^2^, 20.0–21.0 kg/m^2^, 21.0–22.0 kg/m^2^, 22.0–23.0 kg/m^2^, 23.0–24.0 kg/m^2^, ≥24.0 kg/m^2^). Before the primary analysis, we performed preliminary analysis after categorizing the participants into quartiles (Q1: lowest, Q2, Q3 and Q4: highest) by BMI at college age to see the rough trend of the association between BMI at young age and hypertension. We defined the lowest BMI group (Q1 or BMI<20.0 kg/m^2^) as the reference category. The cumulative incidence rate of hypertension according to the BMI categories was compared by χ^2^ test. Hazards ratios and 95 percent confidence interval (95% CI) relating BMI to risk of hypertension were constructed with the Cox proportional hazards analysis. The unadjusted and multivariable-adjusted relative risks were computed. In the Model 1, the adjusted data included age (continuous variable, 1-year-old ticks), year of graduation (continuous variable, 1-year increments), and smoking (category, yes or no). In addition, several factors in middle age, such as low physical activity and over nutrition were reported as risk factor of elevated blood pressure [8, 21–26]. Thus, the model 2 further included current exercise status (continuous variable, 1-met*hour/week increments) and current dietary intake (continuous variable, kcal/day increments) as confounding factor. The year of graduation was used as surrogate variables of economic status. All statistical analyses were conducted using SPSS version 23.0 for Windows software (SPSS Inc., Chicago, IL).

## Results

### Characteristics of study participants

Before the primary analysis, we performed preliminary analysis after categorizing the participants into quartiles (Q1: lowest, Q2, Q3 and Q4: highest) by BMI at college age (Table 1). Median age at graduated college were 22 (IQR: 22–22) years old. The median BMIs at college age were Q1: 20.4 kg/m^2^ (interquartile range: IQR: 19.7–20.7, n = 159), Q2: 21.5 (21.3–21.8, n = 159), Q3: 22.4 (22.2–22.7, n = 159) and Q4: 23.9 (23.4–25.0, n = 159). The median BMI for all study participants and for each category were within the normal range, while 36 participants (5.7%) were overweight (BMI ≥25 to <30 kg/m^2^) and only five (0.8%) were obese (BMI ≥30 kg/m^2^). Almost all of the participants had participated in a college sports club. Highest BMI group (Q4) was high percentage of individuals who participated in power sports, on the other hand, lowest BMI group (Q1) was high percentage of individuals who participated in endurance sports. The percentages of smokers was comparable among the groups.

**Table 1:** Baseline characteristics of study subjects according to BMI in former male college athletes.

	Q1 (n = 159)	Q2 (n = 159)	Q3 (n = 159)	Q4 (n = 159)	All (n = 636)
Age, years	22 (22–22)	22 (22–22)	22 (22–22)	22 (22–22)	22 (22–22)
Year of graduation	1982 (1978–1985)	1982 (1978–1986)	1983 (1979–1986)	1982 (1978–1986)	1982 (1978–1986)
Body Mass Index, kg/m^2^	20.4 (19.7–20.7)	21.5 (21.3–21.8)	22.4 (22.2–22.7)	23.9 (23.4–25.0)	22.0 (21.1–22.9)
Body Height, cm	172.3 (167.6–175.8)	171.8 (167.6–177.9)	172.3 (168.2–177.5)	173.3 (168.4–178.0)	172.4 (168.0–177.0)
Body Weight, kg	59.9 (57.1–62.6)	63.5 (60.5–67.9)	66.5 (63.2–70.5)	72.6 (67.8–77.5)	65.1 (61.0–70.4)
Smoker, n (%)	74 (47.8)	77 (48.4)	83 (52.2)	73 (45.9)	309 (48.6)
College sports club participation, n (%)	158 (99.4)	154 (96.9)	156 (98.1)	158 (99.4)	626 (98.4)
Endurance sports, n (%)	32 (20.1)	10 (6.3)	8 (5.0)	6 (3.8)	56 (8.8)
Mixed sports, n (%)	109 (68.6)	126 (79.2)	140 (88.1)	97 (61.0)	472 (74.2)
Power sports, n (%)	2 (1.3)	7 (4.4)	3 (1.9)	46 (28.9)	58 (9.1)
No-participation / Unknown, n (%)	16 (10.1)	16 (10.1)	8 (5.0)	10 (6.3)	50 (7.9)

Data at time of college and are median (IQR: interquartile range), number (%), or column percentage.

### Association between BMI and incidence of hypertension

The follow-up period of this study was 27-year (interquartile range: IQR: 23–31 years), which included 17,059 person-years of observation. The median age at the time of the follow-up questionnaire was 49 years (IQR: 45–53 years), where 120 men had developed hypertension, with unadjusted cumulative incidence of hypertension of 18.9%.

**Fig 1:**
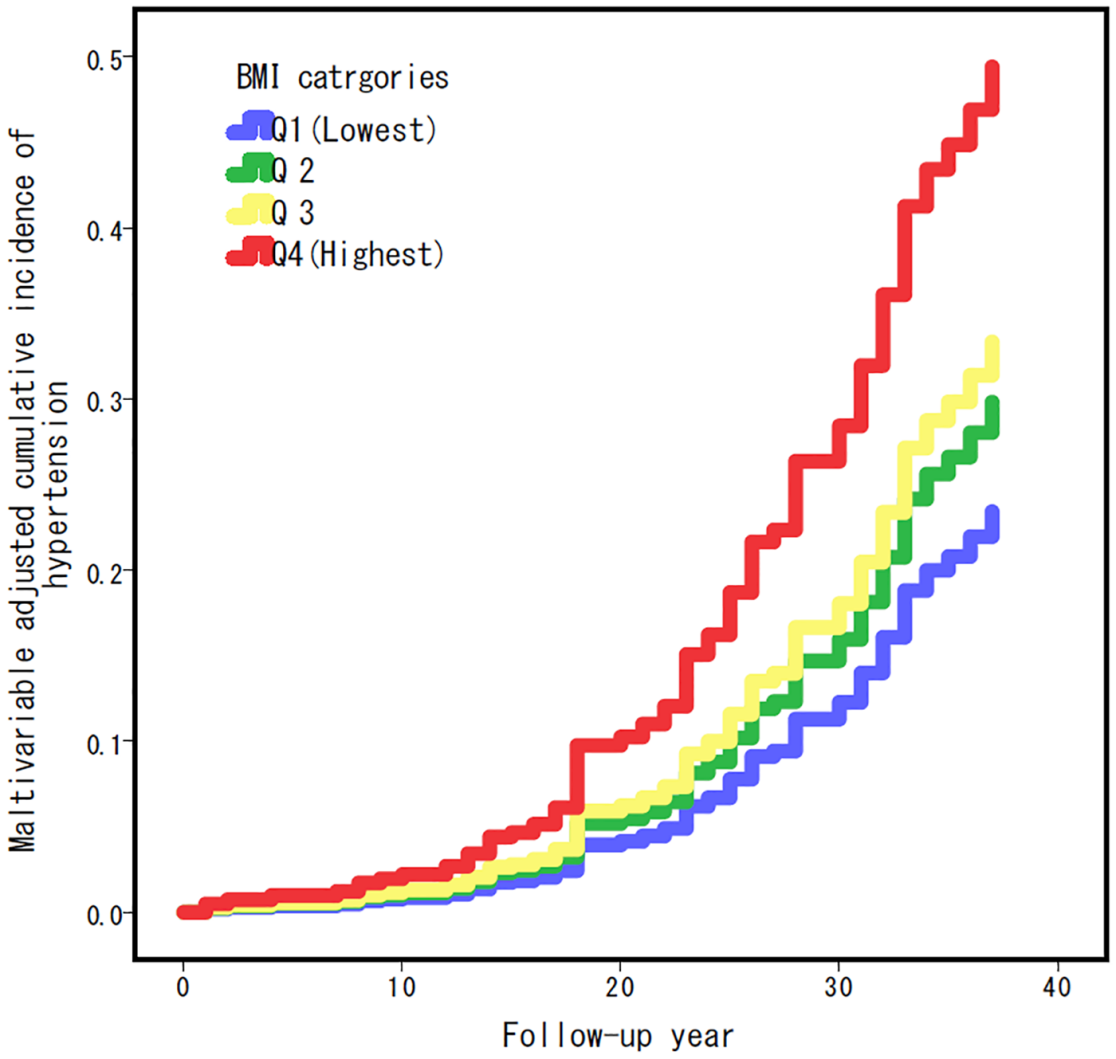
Multivariable adjusted cumulative incidence rate curve for hypertension during the follow-up, according to BMI (quartile).

To examine the relationship between BMI and development of hypertension, we evaluated the rate of incidence and hazard ratios in the Q1 (lowest BMI group; reference), Q2, Q3 and Q4 (highest BMI group). The prevalence rates of hypertension in Q1 to Q4 were significantly different among the groups, the highest BMI group included the highest rate of hypertension. Compared with men in the lowest group of BMI (Q1), men in the highest group of BMI (Q4) were at 2.5 times increased risk of hypertension (Table 2). The trend was persistently seen even after adjustment for age, year of graduation, smoking, current exercise status and current dietary intake (Table 2). In addition, the multivariable adjusted cumulative incidence rate of hypertension was associated with higher BMI throughout the follow-up (Fig 1). These results suggest a significant association between BMI at a young age and future development of hypertension in Japanese men.

**Table 2:** Hazard ratio for hypertension according to BMI (quartiles).

	BMI, quartiles				p for Trend
	Q1 (n = 159)	Q2 (n = 159)	Q3 (n = 159)	Q4 (n = 159)	
Follow-up year	28 (25–32)	27 (23–31)	27 (23–31)	27 (22–31)	
Person-years of follow-up	4435	4218	4248	4158	
Diagnosed with hypertension (n, %)	21 (13.2)	24 (15.3)	28 (17.6)	47 (29.6)	0.001
Incidence per 10,000 parson-years	47.4	56.9	65.9	113.0	
Unadjusted hazard ratio (95% CI)	1.00	1.30 (0.72–2.33)	1.50 (0.85–2.64)	2.56 (1.53–4.29)	<0.001
Multivariable adjusted hazard ratio* (95% CI) Model 1	1.00	1.33 (0.74–2.39)	1.52 (0.86–2.68)	2.55 (1.53–4.27)	<0.001
Multivariable adjusted hazard ratio* (95% CI) Model 2	1.00	1.15 (0.63–2.11)	1.44 (0.81–2.55)	2.52 (1.51–4.22)	<0.001
Age, years	0.95 (0.62–1.47)				0.829
Year of graduation	0.99 (0.95–1.04)				0.756
Smoker (yes/no)	0.69 (0.47–0.99)				0.047
Current exercise status (mets*houre/week)	0.99 (0.98–1.00)				0.187
Current dietary intake (kcal/day)	1.00 (1.00–1.00)				0.988

*Cox proportional hazards models adjusted for model 1: baseline characteristics (age, year of graduation and smoking), model 2: current exercise status and current dietary intake with model 1.

**Fig 2:**
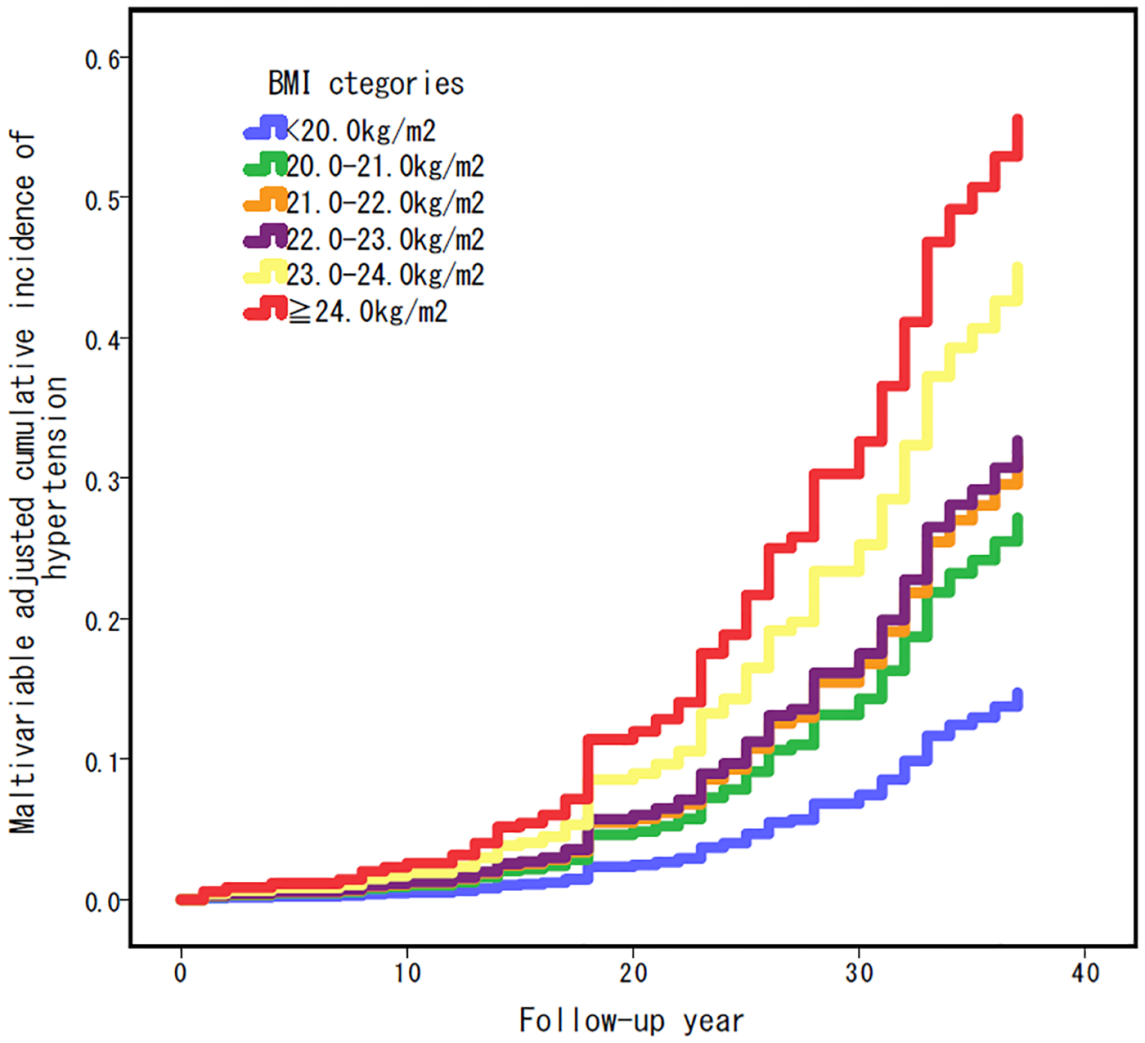
Multivariable adjusted cumulative incidence rate curve for hypertension during the follow-up, according to BMI (six categories).

Then, we re-analyzed by six categories as primary analysis (Table 3). The incidence of hypertension per 10,000 person-years was associated with BMI at young age, the highest BMI category was highest incidence (Table 4). Compared with men in the lowest BMI category (BMI<20.0 kg/m^2^), men in the BMI category of 23-24kg/m^2^ or over 24kg/m^2^ had ~3 or ~4 times higher risk of hypertension, respectively (Table 4). The trend was persistently seen even after adjustment for age, year of graduation, smoking, current exercise status and current dietary intake (Table 4 and Fig 2).

**Table 3:** Baseline characteristics of study subjects according to BMI in former male college athletes.

	<20kg/m^2^ (n = 53)	20-21kg/m^2^ (n = 103)	21-22kg/m^2^ (n = 163)	22-23kg/m^2^ (n = 166)	23-24kg/m^2^ (n = 76)	≥24kg/m^2^ (n = 75)
Age, years	22 (22–22)	22 (22–22)	22 (22–22)	22 (22–22)	22 (22–22)	22 (22–22)
Year of graduation	1982 (1977–1985)	1982 (1979–1985)	1982 (1978–1986)	1982 (1979–1986)	1981 (1977–1985)	1983 (1979–1987)
Body Mass Index, kg/m^2^	19.4 (19.1–19.8)	20.6 (20.4–20.8)	21.5 (21.3–21.8)	22.4 (22.2–22.7)	23.5 (23.2–23.7)	25.2 (24.3–26.1)
Body Height, cm	171.6 (166.8–176.5)	172.6 (169.1–175.3)	171.8 (167.5–177.9	172.4 (168.4–177.5)	173.4 (167.6–176.7)	173.3 (168.6–180.1)
Body Weight, kg	57.3 (53.2–60.3)	61.2 (58.7–63.4)	63.5 (60.3–67.9)	66.6 (63.4–70.6)	70.4 (65.7–73.1)	76.4 (70.8–83.0)
Smoker, n (%)	19 (35.8)	55 (53.4)	80 (49.1)	87 (52.4)	37 (48.7)	31 (41.3)
College sports club participation, n (%)	53 (100)	102 (99.0)	158 (96.9)	163 (98.2)	74 (100)	74 (98.7)
Endurance sports, n (%)	17 (32.1)	15 (14.6)	10 (6.1)	8 (4.8)	4 (5.3)	2 (2.7)
Mixed sports, n (%)	32 (60.4)	74 (71.8)	130 (79.8)	145 (87.3)	55 (72.4)	36 (48.0)
Power sports, n (%)	1 (1.9)	1 (1.0)	7 (4.3)	4 (2.4)	12 (15.8)	33 (44.0)
No-participation / Unknown, n (%)	3 (5.7)	13 (12.6)	16 (9.8)	9 (5.4)	5 (6.6)	4 (5.3)

Data at time of college and are median (IQR: interquartile range), number (%), or column percentage.

**Table 4:** Hazard ratio for hypertension according to BMI (six categories).

	BMI at college age						p for Trend
	<20kg/m^2^ (n = 53)	20-21kg/m^2^ (n = 103)	21-22kg/m^2^ (n = 163)	22-23kg/m^2^ (n = 166)	23-24kg/m^2^ (n = 76)	≥24kg/m^2^ (n = 75)	
Follow-up year	29.0 (24.5–33.5)	28.0 (25.0–32.0)	27.0 (23.0–31.0)	27.0 (23.0–31.0)	28.0 (23.3–32.0)	24.0 (21.0–30.3)	
Person-years of follow-up	1542	2828	4306	4453	2099	1831	
Diagnosed with hypertension (n, %)	5 (9.4)	15 (14.6)	26 (16.1)	29 (17.5)	23 (30.3)	22 (29.3)	<0.001
Incidence per 10,000 parson-years	32.4	53.0	60.4	65.1	109.6	120.2	
Unadjusted hazard ratio (95% CI)	1.00	1.80 (0.65–4.94)	2.17 (0.83–5.64)	2.29 (0.89–5.92)	3.60 (1.37–9.47)	4.72 (1.78–12.48)	<0.001
Multivariable adjusted hazard ratio* (95% CI) Model 1	1.00	1.99 (0.72–5.51)	2.37 (0.91–6.20)	2.49 (0.96–6.44)	3.76 (1.43–9.89)	5.09 (1.92–13.51)	<0.001
Multivariable adjusted hazard ratio* (95% CI) Model 2	1.00	1.98 (0.72–5.49)	1.99 (0.75–5.27)	2.44 (0.94–6.32)	3.73 (1.42–9.83)	4.92 (1.85–13.04)	<0.001
Age, years	0.95 (0.62–1.46)						0.800
Year of graduation	0.99 (0.94–1.04)						0.668
Smoker (yes/no)	0.68 (0.47–0.99)						0.043
Current exercise status (mets*houre/week)	0.99 (0.98–1.00)						0.200
Current dietary intake (kcal/day)	1.00 (1.00–1.00)						0.992

* Cox proportional hazards models adjusted for model 1: baseline characteristics (age, year of graduation and smoking and college sports club participation), model 2: current exercise status and dietary intake with model 1.

## Discussion

In the present study, we investigated the relationship between BMI at young age and future development of hypertension in University-educated Japanese men. After adjustment for potential confounding factors, the results identified BMI category of 23–24 kg/m^2^ or over 24 kg/m^2^ were ~3 or ~4 times high risk for hypertension compared with BMI category of under 20 kg/m^2^. These data suggest that a slightly high BMI at a young age, even when within the "normal range", may influence future development of hypertension in Japanese men.

Our results demonstrated that for Japanese male college athletes, slightly increased BMI at young age is a risk factor for future hypertension. Median BMI of this study participants was 22.0kg/m^2^ (IQR: 21.1–22.9kg/m^2^), while only 37 participants (5.8%) were overweight (BMI ≥25.0 to <30.0 kg/m^2^) and only five (0.8%) were obese (BMI ≥30.0 kg/m^2^). In these subjects, we found BMI ≥23kg/m^2^ at young age might be cut-off value of increased risk of future hypertension. This cut-off value was similar to previous report showing that subjects with BMI ≥23kg/m^2^ at 20 years old have ~2 times higher risk of hypertension in middle age, compared with subjects with BMI <23.0 kg/m^2^ [16].

What is the mechanism through which the normal BMI at young increases the chance of hypertension in later life? Although our study did not investigate the exact mechanism, background data may help in the interpretation of our results. At least in adults, the body composition profile of Asians differs from that of Caucasians and African-Americans. Asians have a higher body fat content and more visceral adipose tissue, compared with Caucasians and African Americans of similar BMI [11, 27, 28]. Furthermore, previous studies reported the presence of muscle insulin resistance in non-obese subjects with one or more cardiometabolic risk factors [29]. It was also demonstrated that muscle insulin sensitivity is significantly associated with hypertension in non-obese subjects[29] and that insulin sensitivity is a risk factor for hypertension[30].

The present study has several limitations. First, the participants are not representative of all Japanese men and small cohorts. The study subjects were alumni of only one university department and almost all subjects were former college athlete, although they came from all over Japan. In addition, female alumni were not included because they were not registered at the college in 1971. Second, alumni without data of either the physical examination while at college or follow-up questionnaire were excluded. These exclusions limited the generalization of the results. Third, self-selection bias could have occurred because the participants who answered the follow-up questionnaires may have been those who were the healthiest. Finally, recall bias was possible because the questionnaire was cross-sectional and participants needed to recall their medical background. However, our previous studies have used the same method[18–20] and established the validity of the self-reports examination by re-examination[31]. In addition, in Japan, most people undergo free medical screening by a physician at least once annually at work or school.

## Conclusion

In conclusion, our data suggested that the slightly increased BMI at young age is a significant risk factor for future hypertension in Japanese men.

## References

[1] Adams KF, Schatzkin A, Harris TB, Kipnis V, Mouw T, Ballard-Barbash R, et al Overweight, obesity, and mortality in a large prospective cohort of persons 50 to 71 years old. The New England journal of medicine. 2006;355(8):763–78. doi: 10.1056/NEJMoa055643 . 16926275 10.1056/NEJMoa055643

[2] Wu CY, Chou YC, Huang N, Chou YJ, Hu HY, Li CP. Association of body mass index with all-cause and cardiovascular disease mortality in the elderly. PloS one. 2014;9(7):e102589 doi: 10.1371/journal.pone.0102589 25014070 10.1371/journal.pone.0102589 PMC4094563

[3] Barrett-Connor EL. Obesity, atherosclerosis, and coronary artery disease. Annals of internal medicine. 1985;103(6 (Pt 2)):1010–9. . 3904565 10.7326/0003-4819-103-6-1010

[4] Talavera-Garcia E, Delgado-Lista J, Garcia-Rios A, Delgado-Casado N, Gomez-Luna P, Gomez-Garduno A, et al Influence of Obesity and Metabolic Disease on Carotid Atherosclerosis in Patients with Coronary Artery Disease (CordioPrev Study). PloS one. 2016;11(4):e0153096 doi: 10.1371/journal.pone.0153096 27064675 10.1371/journal.pone.0153096 PMC4827867

[5] Hwang LC, Tsai CH, Chen TH. Overweight and obesity-related metabolic disorders in hospital employees. J Formos Med Assoc. 2006;105(1):56–63. doi: 10.1016/S0929-6646(09)60109-1 . 16440071 10.1016/S0929-6646(09)60109-1

[6] Boffetta P, McLerran D, Chen Y, Inoue M, Sinha R, He J, et al Body mass index and diabetes in Asia: a cross-sectional pooled analysis of 900,000 individuals in the Asia cohort consortium. PloS one. 2011;6(6):e19930 doi: 10.1371/journal.pone.0019930 21731609 10.1371/journal.pone.0019930 PMC3120751

[7] Shihab HM, Meoni LA, Chu AY, Wang NY, Ford DE, Liang KY, et al Body mass index and risk of incident hypertension over the life course: the Johns Hopkins Precursors Study. Circulation. 2012;126(25):2983–9. doi: 10.1161/CIRCULATIONAHA.112.117333 23151344 10.1161/CIRCULATIONAHA.112.117333 PMC3743236

[8] Crump C, Sundquist J, Winkleby MA, Sundquist K. Interactive Effects of Physical Fitness and Body Mass Index on the Risk of Hypertension. JAMA Intern Med. 2016;176(2):210–6. doi: 10.1001/jamainternmed.2015.7444 26784837 10.1001/jamainternmed.2015.7444 PMC4803286

[9] Tirosh A, Afek A, Rudich A, Percik R, Gordon B, Ayalon N, et al Progression of normotensive adolescents to hypertensive adults: a study of 26,980 teenagers. Hypertension. 2010;56(2):203–9. doi: 10.1161/HYPERTENSIONAHA.109.146415 . 20547973 10.1161/HYPERTENSIONAHA.109.146415

[10] Ng M, Fleming T, Robinson M, Thomson B, Graetz N, Margono C, et al Global, regional, and national prevalence of overweight and obesity in children and adults during 1980–2013: a systematic analysis for the Global Burden of Disease Study 2013. Lancet. 2014;384(9945):766–81. doi: 10.1016/S0140-6736(14)60460-8 24880830 10.1016/S0140-6736(14)60460-8 PMC4624264

[11] Pan WH, Flegal KM, Chang HY, Yeh WT, Yeh CJ, Lee WC. Body mass index and obesity-related metabolic disorders in Taiwanese and US whites and blacks: implications for definitions of overweight and obesity for Asians. The American journal of clinical nutrition. 2004;79(1):31–9. . 14684394 10.1093/ajcn/79.1.31

[12] Gallagher D. Overweight and obesity BMI cut-offs and their relation to metabolic disorders in Koreans/Asians. Obesity research. 2004;12(3):440–1. doi: 10.1038/oby.2004.48 . 15044659 10.1038/oby.2004.48

[13] Hsu WC, Araneta MR, Kanaya AM, Chiang JL, Fujimoto W. BMI cut points to identify at-risk Asian Americans for type 2 diabetes screening. Diabetes care. 2015;38(1):150–8. doi: 10.2337/dc14-2391 25538311 10.2337/dc14-2391 PMC4392932

[14] Kim Y, Suh YK, Choi H. BMI and metabolic disorders in South Korean adults: 1998 Korea National Health and Nutrition Survey. Obesity research. 2004;12(3):445–53. doi: 10.1038/oby.2004.50 . 15044661 10.1038/oby.2004.50

[15] Sakuta H, Suzuki T. Overweight male personnel of the Japan Self-Defense Forces with body mass indices of 23.0–24.9 and obesity-related metabolic disorders. Environ Health Prev Med. 2008;13(2):116–20. doi: 10.1007/s12199-007-0010-0 19568890 10.1007/s12199-007-0010-0 PMC2698264

[16] Heianza Y, Kodama S, Arase Y, Hsieh SD, Yoshizawa S, Tsuji H, et al Role of body mass index history in predicting risk of the development of hypertension in Japanese individuals: Toranomon Hospital Health Management Center Study 18 (TOPICS 18). Hypertension. 2014;64(2):247–52. doi: 10.1161/HYPERTENSIONAHA.113.02918 . 24842920 10.1161/HYPERTENSIONAHA.113.02918

[17] Huang Z, Willett WC, Manson JE, Rosner B, Stampfer MJ, Speizer FE, et al Body weight, weight change, and risk for hypertension in women. Annals of internal medicine. 1998;128(2):81–8. . 9441586 10.7326/0003-4819-128-2-199801150-00001

[18] Someya Y, Kawai S, Marui E, Takata K. Influences of exercise and eating habits during student age on lifestyle-related diseases in middle age, based on the male alumni of Juntendo University. Journal of health and sports science Juntendo. 2010;1(3):421–5.

[19] Someya Y, Kawai S, Kohmura Y, Aoki K, Daida H. Cardiorespiratory fitness and the incidence of type 2 diabetes: a cohort study of Japanese male athletes. BMC public health. 2014;14:493 Epub 2014/06/03. doi: 10.1186/1471-2458-14-493 24885699 10.1186/1471-2458-14-493 PMC4038597

[20] Someya Y, Tamura Y, Kohmura Y, Aoki K, Kawai S, Daida H. Muscle strength at young age is not associated with future development of type 2 diabetes in Japanese male athletes. The Journal of Physical Fitness and Sports Medicine. 2017;6(3):167–73. doi: 10.7600/jpfsm.6.167

[21] Gelber RP, Gaziano JM, Manson JE, Buring JE, Sesso HD. A prospective study of body mass index and the risk of developing hypertension in men. Am J Hypertens. 2007;20(4):370–7. doi: 10.1016/j.amjhyper.2006.10.011 17386342 10.1016/j.amjhyper.2006.10.011 PMC1920107

[22] Hu G, Barengo NC, Tuomilehto J, Lakka TA, Nissinen A, Jousilahti P. Relationship of physical activity and body mass index to the risk of hypertension: a prospective study in Finland. Hypertension. 2004;43(1):25–30. doi: 10.1161/01.HYP.0000107400.72456.19 . 14656958 10.1161/01.HYP.0000107400.72456.19

[23] Hayashi T, Tsumura K, Suematsu C, Okada K, Fujii S, Endo G. Walking to work and the risk for hypertension in men: the Osaka Health Survey. Annals of internal medicine. 1999;131(1):21–6. . 10391811 10.7326/0003-4819-131-1-199907060-00005

[24] Lee JS, Auyeung TW, Leung J, Kwok T, Leung PC, Woo J. Physical frailty in older adults is associated with metabolic and atherosclerotic risk factors and cognitive impairment independent of muscle mass. The journal of nutrition, health & aging. 2011;15(10):857–62. . 22159773 10.1007/s12603-011-0134-1 PMC12878010

[25] Ishikawa-Takata K, Ohta T, Moritaki K, Gotou T, Inoue S. Obesity, weight change and risks for hypertension, diabetes and hypercholesterolemia in Japanese men. European journal of clinical nutrition. 2002;56:601–7. doi: 10.1038/sj.ejcn.1601364 12080398 10.1038/sj.ejcn.1601364

[26] Chandra Alvin, Neeland Ian J., Berry Jarett D., Ayers Colby R., Rohatgi Anand, Das Sandeep R., et al The relationship of body mass and fat distribution with incident hypertension. JACC. 2014;64(10):997–1002. doi: 10.1016/j.jacc.2014.05.057 25190234 10.1016/j.jacc.2014.05.057

[27] Ito H, Nakasuga K, Ohshima A, Maruyama T, Kaji Y, Harada M, et al Detection of cardiovascular risk factors by indices of obesity obtained from anthropometry and dual-energy X-ray absorptiometry in Japanese individuals. International journal of obesity and related metabolic disorders: journal of the International Association for the Study of Obesity. 2003;27(2):232–7. doi: 10.1038/sj.ijo.802226 . 12587004 10.1038/sj.ijo.802226

[28] Chang CJ, Wu CH, Chang CS, Yao WJ, Yang YC, Wu JS, et al Low body mass index but high percent body fat in Taiwanese subjects: implications of obesity cutoffs. International journal of obesity and related metabolic disorders: journal of the International Association for the Study of Obesity. 2003;27(2):253–9. doi: 10.1038/sj.ijo.802197 . 12587007 10.1038/sj.ijo.802197

[29] Takeno K, Tamura Y, Kawaguchi M, Kakehi S, Watanabe T, Funayama T, et al Relation between insulin sensitivity and metabolic abnormalities in Japanese men with BMI of 23–25 kg/m2. The Journal of clinical endocrinology and metabolism. 2016:jc20161650. doi: 10.1210/jc.2016-1650 . 27383116 10.1210/jc.2016-1650

[30] Hirose H, Saito I, Kawabe H, Saruta T. Insulin resistance and hypertension: seven-year follow-up study in middle-aged Japanese men (the KEIO study). Hypertens Res. 2003;26(10):795–800. . 14621182 10.1291/hypres.26.795

[31] Paffenbarger RS Jr., Wing AL. Chronic disease in former college students. XII. Early precursors of adult-onset diabetes mellitus. American journal of epidemiology. 1973;97(5):314–23. . 4701675 10.1093/oxfordjournals.aje.a121511

